# Functional Application of Noble Metal Nanoparticles In Situ Synthesized on Ramie Fibers

**DOI:** 10.1186/s11671-015-1074-1

**Published:** 2015-09-17

**Authors:** Bin Tang, Ya Yao, Jingliang Li, Si Qin, Haijin Zhu, Jasjeet Kaur, Wu Chen, Lu Sun, Xungai Wang

**Affiliations:** School of Textile Science and Engineering, Wuhan Textile University, Wuhan, 430073 China; Institute for Frontier Materials, Deakin University, Geelong, VIC 3216 Australia

**Keywords:** Ramie, Silver nanoparticle, Gold nanoparticle, In situ synthesis, Coloration, Catalysis

## Abstract

Different functions were imparted to ramie fibers through treatment with noble metal nanoparticles including silver and gold nanoparticles. The in situ synthesis of silver and gold nanoparticles was achieved by heating in the presence of ramie fibers in the corresponding solutions of precursors. The unique optical property of synthesized noble metal nanoparticles, i.e., localized surface plasmon resonance, endowed ramie fibers with bright colors. Color strength (K/S) of fibers increased with heating temperature. Silver nanoparticles were obtained in alkaline solution, while acidic condition was conducive to gold nanoparticles. The optical properties of treated ramie fibers were investigated using UV-vis absorption spectroscopy. Scanning electron microscopy (SEM) was employed to observe the morphologies of silver and gold nanoparticles in situ synthesized on fibers. The ramie fibers treated with noble metal nanoparticles showed remarkable catalytic activity for reduction of 4-nitrophenol (4-NP) by sodium borohydride. Moreover, the silver nanoparticle treatment showed significant antibacterial property on ramie fibers.

**Electronic supplementary material:**

The online version of this article (doi:10.1186/s11671-015-1074-1) contains supplementary material, which is available to authorized users.

## Background

Functionalization of textiles with nanoparticles has attracted intensive attentions of scientists and engineers in recent years. A number of strategies have been developed to enhance functions of textiles [1–4], such as antibacterial treatment [1, 5], self-cleaning coating [6, 7], and UV protection enhancement [8, 9]. Semiconductor (e.g., TiO_2_ and ZnO) nanoparticles have been applied to textiles for functions such as UV-blocking and self-cleaning [10, 11]. Silver nanoparticle as a broad antibacterial agent is widely used for antibacterial finishing of textiles [12, 13]. Besides antibacterial property, noble metal nanoparticles (e.g., silver and gold) possess particular localized surface plasmon resonance (LSPR) property, which leads to bright colors. Some strategies have been developed to utilize noble metal nanoparticles to functionalize different fibers such as cotton, wool, and silk. The coloration of fibers has been achieved through treatment with silver nanoparticles, taking advantage of the LSPR optical property of noble metal nanoparticles [5, 14, 15]. Similarly, gold nanoparticles can also endow fabrics with different functions including colors, UV protection, and antibacterial properties [16–18].

Ramie from a perennial herbaceous plant of the Urticaceae family is one of the most important bast plant fibers and has a silky luster. It is superior to cotton and silk in length and strength [19–21]. Ramie fibers have been applied to an increasing number of textile products including canvas, clothing fabrics, towels, cordages, and garments, due to their high tenacity, excellent thermal conductivity, good comfort, and significant tensile properties [21–23]. China and several other Asian countries are main producing areas of ramie fiber [24, 25]. Functional modification of ramie fibers is one way to promote product value and satisfy the increasing needs from consumers. Regarding modifications of ramie, Zheng et al. coated ramie fabrics with titanium dioxide (TiO_2_) nanoparticles via a dip-pad-dry process [26]. The ramie fabrics treated with TiO_2_ showed antibacterial and UV-protective properties. However, the research focused on the functionalization of ramie with nanoparticles is very limited.

In this study, the functionalization of ramie fibers was realized through in situ synthesis of silver and gold nanoparticles. The silver nanoparticle-treated ramie fiber displayed a yellow color, while gold nanoparticles endowed the ramie fiber with red and purple colors. The changes in color strength (K/S) of ramie fibers with pH value of solution were also observed. Antibacterial property of the ramie fibers treated with silver nanoparticles was evaluated. The color fastness to washing of the treated ramie was tested. Moreover, the catalytic ability of ramie fibers treated with silver and gold nanoparticles was determined using reduction of 4-nitrophenol by sodium borohydride as a model reaction.

## Methods

### Materials

AgNO_3_ (>99 %), tetrachloroauric (III) acid (HAuCl_4_ · 3H_2_O, >99 %), NaOH (≥97.0 %), acetic acid (>99.7 %), 4-nitrophenol (≥99 %), and sodium borohydride (>98 %) were purchased from Sigma-Aldrich. All chemicals were analytic grade reagents and used without further purification. Ramie fibers and fabrics were kindly provided by Engineering Research Center for Clean Production of Textile Printing in Wuhan Textile University. They were used without pretreatment.

### Instruments

The UV-vis diffuse reflectance absorption spectra of fibers were recorded by a Varian Cary 5000 UV-VIS-NIR spectrophotometer with a diffuse reflectance accessory (DRA-2500). Scanning electron microscopy (SEM) measurements were performed with a Supra 55 VP field emission SEM. The color strength (K/S) of ramie fibers/fabrics with noble metal nanoparticles was calculated using the Kubelka–Munk equation as follows:

$$ K/S=\frac{{\left(1-R\right)}^2}{2R} $$

where *K* is the absorption coefficient of the substrate, *S* is the scattering coefficient of the substrate, and *R* is the reflectance of the fibers/fabrics at maximum absorption, measured using a Datacolor Spectraflash SF600 Plus-CT spectrophotometer. Heating reaction was performed in a Ratek shaking water bath. A Varian AA-140 atomic absorption spectrophotometer was employed to analyze the concentration of silver and gold ions.

### In Situ Synthesis of Silver and Gold Nanoparticles on Ramie Fibers

Ramie fibers were washed for 3 min with hot water (70 °C) followed by rinsing with deionized water at room temperature. The washed fibers were immersed in precursor solutions of metal nanoparticles (AgNO_3_ for silver nanoparticles and HAuCl_4_ for gold nanoparticles) with different concentrations. The weight ratio of aqueous solution to fibers was 80. The ramie fibers were immersed in precursor solutions for 15 min at room temperature and the pH values of the solutions were adjusted. After that, the solutions were heated at different temperatures for 60 min in an oscillating water bath. The treated fibers were rinsed with running deionized water and dried at room temperature. The details of the corresponding experimental conditions for in situ synthesis of silver and gold nanoparticles are listed in Additional file 1: Tables S1 and Additional file 1: S2, respectively.

### Color Fastness to Washing

Washing fastness was evaluated in accordance with Australian Standard AS 2001.4.15—2006. The ramie fabrics treated with silver or gold nanoparticles were washed for 45 min at 50 °C in the presence of ECE reference detergent (4 g/L) by using a lab dyeing machine (Ahiba, Top Speed Nuance). The CIE Lab color coordinate values (L*, a*, and b*) for each specimen were measured before and after washing. L* represents the lightness/darkness, a* value represents the red or green chroma, and b* represents the chromaticity coordinate for yellow/blue. The color difference (ΔE) was obtained based on the changes in color coordinates (ΔL*, Δa*, and Δb*) with the formula: ΔE = [(ΔL*)^2^ + (Δa*)^2^ + (Δb*)^2^]^1/2^. The color difference (ΔE) of ramie fabrics before and after washing was measured by spectrophotometer to assess washing fastness of ramie fabrics according to Australian Standard AS 2001.4.A05—2004.

### Catalytic Activity

To investigate the catalytic efficiency and reusability of the ramie fibers treated with noble metal nanoparticles, the catalytic conversion of 4-nitrophenol into 4-aminophenol by sodium borohydride (NaBH_4_) was performed in the presence of untreated and treated ramie. In a typical experiment, 1.0 mL of NaBH_4_ solution (3.42 M) was added into 40 mL of 4-nitrophenol aqueous solution (0.025 mM). Subsequently, 0.0485 g of untreated ramie fibers or treated ramie fibers (Ag-90-10-1, Ag-90-10-3 and Au-90-5-6) was added into the mixing solution of 4-nitrophenol and NaBH_4_ under vigorous stirring. UV–visible absorption spectra were monitored during the conversion of 4-nitrophenol into 4-aminophenol.

### Antibacterial Test Against Gram-Negative Bacteria

Gram-negative bacteria, *Escherichia coli* (*E. coli*) (ATCC 11229), were used as test organisms. Antibacterial test was performed on untreated and silver nanoparticle-treated ramie fibers. The antibacterial test was carried out according to the AATCC 100-2004 (Clause 10.2) test standard with slight modifications. Fifty microliters of bacteria were added to treated samples in separate flasks. After 1.0 min, 50 mL of sterile deionized water was poured into each flask, followed by vigorous shaking. Then, the flasks were incubated for 18 h in a shaker oven at 120 rpm and 37 °C. After that, the fiber samples were collected and the solution left in the flask was further diluted to get countable number of bacterial colonies. A 10^3^ dilution was suitable for obtaining colonies between 30 and 300. One hundred microliters of the 10^3^ dilution obtained was placed on the nutrient agar plates. These plates were then incubated for 18 h in an oven at 37 °C. The bacterial activity was recorded by photography. The test was carried out in triplicate and the entire experiments were repeated three times.

## Results and Discussion

**Fig. 1:**
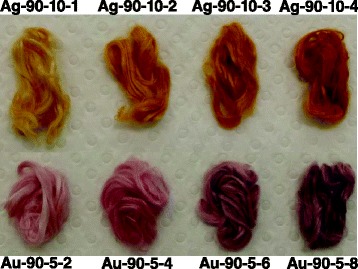
Photographs of ramie fibers with in situ synthesized silver and gold nanoparticles

**Fig. 2:**
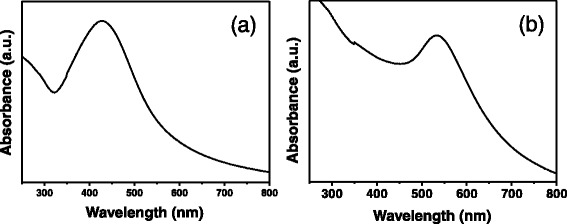
UV-vis reflectance absorption spectra of ramie fibers treated with **a** silver (Ag-90-10-3) and **b** gold nanoparticles (Au-90-5-6)

**Fig. 3:**
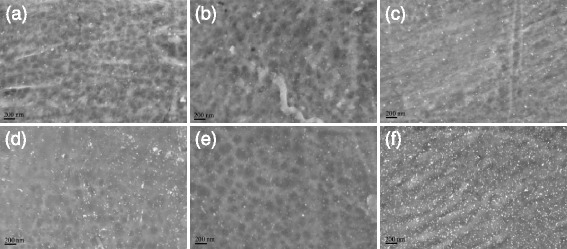
SEM images of ramie fibers treated with silver and gold nanoparticles corresponding to **a** Ag-90-10-2, **b** Ag-90-10-3, **c** Ag-90-10-4, **d** Au-90-5-4, **e** Au-90-5-6, and **f** Au-90-5-8

Yellow ramie fibers were obtained after in situ synthesis of silver nanoparticles (Fig. 1). The color of ramie fibers treated with silver nanoparticles darkened as the concentration of silver ions increased. Whereas, in situ synthesis of gold nanoparticles imparted ramie fiber with red and purple colors (Fig. 1). The color changed to purple from red when the concentration of gold ions increased. The colors generated from unique optical property of noble metal nanoparticles formed on ramie fibers. To investigate the optical features of treated ramie fibers, the UV-vis reflectance absorption spectra of ramie fibers with nanoparticles were measured. Absorption spectrum of fibers treated with silver nanoparticles displays a single band centered at 428 nm, which is due to LSPR of silver nanoparticles (Fig. 2a). The ramie fibers treated by gold nanoparticles presented an UV-vis reflectance absorption band around 533 nm assigned to LSPR of gold nanoparticles (Fig. 2b). The LSPR optical property led to the bright color of fibers after treatment, which realized the coloration of ramie without using traditional dyes. SEM characterizations were performed to observe the synthesis of silver and gold nanoparticles on ramie. SEM images corresponding to different concentrations of silver ions are shown in Fig. 3a–c. Spherical silver nanoparticles can be seen on the surface of ramie fibers, without visible aggregation, revealing silver nanoparticles were formed through reduction of silver ions by ramie fibers. The gold nanoparticles were seen on ramie fiber with different concentrations of gold ions as well (Fig. 3d–f). The SEM images of treated fibers further testify the presence of silver and gold nanoparticles on ramie at different pH values. The factors affecting in situ synthesis of silver and gold nanoparticles are discussed in detail hereinafter.

**Fig. 4:**
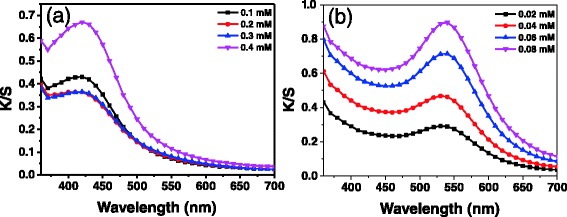
**a** K/S curves of ramie fibers with silver nanoparticles obtained with pH = 10 at 90 °C corresponding to different concentrations of AgNO_3_. **b** K/S curves of ramie fibers with gold nanoparticles obtained with pH = 5 at 90 °C corresponding to different concentrations of HAuCl_4_

In order to investigate the coloration of ramie with noble metal nanoparticles, K/S curves of the treated fibers were measured. The maximum K/S of silver nanoparticle treated ramie fibers was at a wavelength of around 430 nm (Fig. 4a). Its value increased with an increase in silver ion concentration (Additional file 1: Figure S1a). The maximum K/S of gold nanoparticle ramie fibers corresponding to 0.02 mM of gold ions was located at 530 nm (Fig. 4b). The wavelength of maximum K/S changed to 540 nm as the gold ion concentration increased to 0.08 mM, which may be due to increase in size and amount of nanoparticles on ramie fibers. The maximum K/S value increased as the concentration of gold ions increased (Additional file 1: Figure S1b). These results suggest that the ramie fibers can be colored by in situ synthesized silver and gold nanoparticles, and K/S of fibers can be controlled by changing the concentration of precursor ions.

Heating may promote the in situ synthesis of noble metal nanoparticles in the presence of ramie fibers. To investigate the influence of heating on synthesis of noble metal nanoparticles, different temperatures were tested when other reaction conditions remained unchanged. Additional file 1: Tables S3 and S4 displays the detailed conditions for synthesis of silver and gold nanoparticles. Additional file 1: Figure S2a shows the K/S curves of ramie fibers treated with silver nanoparticles obtained at different temperatures. The result reveals that the silver nanoparticles can be obtained in situ on ramie fibers at a low temperature (40 °C). The K/S value increased as the reaction temperature increased (Additional file 1: Figure S2b). It is found that high temperature led to high color strength (K/S) of ramie fibers. The gold nanoparticles were also prepared on ramie fibers at 40 °C (Additional file 1: Figure S3a). The K/S value increased when the temperature was increased to 70 from 40 °C. Nevertheless, the maximum K/S value changed slightly when the reaction temperature was between 70 ~ 90 °C (Additional file 1: Figure S3b), implying that nearly all the gold ions were reduced at 70 °C. The trend of change in K/S curves of gold nanoparticle-treated ramie fibers was different from that of silver nanoparticle-treated ramie fibers, which may be due to difference in redox potential of silver and gold.

**Fig. 5:**
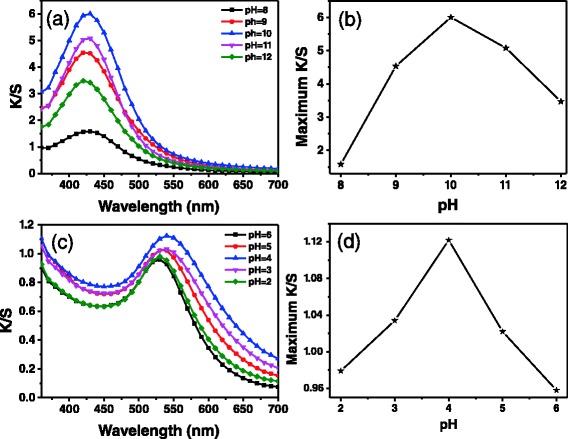
**a** K/S curves of ramie fibers with silver nanoparticles obtained with 0.3 mM of AgNO_3_ at 90 °C under different pH values. **b** Plot of maximum K/S value of silver nanoparticle-treated ramie fibers as a function of pH value. **c** K/S curves of ramie fibers with gold nanoparticles obtained with 0.06 mM of HAuCl_4_ at 90 °C under different pH values. **d** Plot of maximum K/S value of gold nanoparticle-treated ramie fibers as a function of pH value

The silver nanoparticles were in situ synthesized under alkaline condition, while the gold nanoparticles were produced under acidic condition. Ramie fibers were still white after being heated for 3 h at 90 °C when the pH value of the reaction solution was 7 (Additional file 1: Figure S4), revealing no silver nanoparticles were produced at pH = 7. Ramie fibers turned purple in color when the fibers were heated for 3 h at 90 °C in HAuCl_4_ solution with pH = 7 (Additional file 1: Figure S4), which implies there were a few gold nanoparticles synthesized at pH = 7. However, the neutral condition is unfavorable for in situ synthesis of gold nanoparticles on ramie fibers. The K/S curves of silver nanoparticle-treated ramie fibers corresponding to different pH values (8 ~ 12) were measured to investigate the influence of pH on in situ synthesis of silver nanoparticles (Additional file 1: Table S5 and Fig. 5a). The maximum K/S value of ramie fibers at pH = 8 was 1.6. The K/S value increased to 6.0 when the pH value changed to 10 from 8. The increase of K/S indicates that the amount of silver nanoparticles in situ synthesized on fibers increased. Nevertheless, K/S decreased to 3.5 when the pH value increased to 12 from 10 (Fig. 5b). Therefore, the optimal pH value for in situ synthesis of silver nanoparticles should be 10 in this study. Moreover, the synthesis of gold nanoparticles was also investigated at different pH values (2 ~ 6) (Additional file 1: Table S6 and Fig. 5c). The highest K/S value was obtained when the pH vale of solution was 4 (Fig. 5d). However, compared with the fibers with silver nanoparticles, the changes in K/S value of fibers with gold nanoparticles at different pH values were not obvious.

The content of metal in treated ramie fibers was analyzed through measuring the concentration of residual metal ions in reaction solution using atomic absorption spectroscopy (AAS) after the in situ synthesis reactions were finished. Table 1 presents the detailed data about content of silver in the treated ramie. The content of silver in ramie increased as the concentration of the initial AgNO_3_ solution increased (at 90 °C and pH = 10). Regarding the influence of pH value on the content of silver, the samples with identical AgNO_3_ concentration (0.3 mM) and temperature (90 °C) at different pH values were tested. The maximum content of silver was obtained at pH = 10. Compared with the case with pH = 10, the corresponding content of silver decreased obviously when the pH value of solution was adjusted to 7, which implies that alkaline condition improves the content of silver of ramie fibers. The silver content decreased when the pH value was over 10. Moreover, the silver content in ramie under different temperatures was measured. It was found that the content of silver changed little when the temperature was more than 40 °C. Colors of treated ramie fibers arose from the LSPR property of silver nanoparticles in situ synthesized on the fibers. It should be noted that the color strength of treated ramie fibers is related to not only the content of silver but also the shape and size of silver nanoparticles [17, 27]. Besides, the content of gold on ramie fibers treated with gold nanoparticles also increased as the HAuCl_4_ concentration in solution increased as keeping reaction temperature and pH value unchanged (at 90 °C and pH = 5) (Table 2). No gold ions were left in solution when the pH values of reaction system was 2 ~ 4, revealing a low pH value favors absorption of gold ions on ramie fibers (Table 2).

**Table 1:** Content of silver in different ramie fibers treated with silver nanoparticles

Content of Ag (wt%)		Content of Ag (wt%)	
Ag-90-10-1	0.085	Ag-90-9-3	0.176
Ag-90-10-2	0.155	Ag-90-11-3	0.117
Ag-90-10-3	0.233	Ag-90-12-3	0.060
Ag-90-10-4	0.294	Ag-80-10-3	0.228
Ag-90-7-1	0.014	Ag-70-10-3	0.234
Ag-90-7-2	0.029	Ag-60-10-3	0.229
Ag-90-7-3	0.037	Ag-50-10-3	0.214
Ag-90-7-4	0.074	Ag-40-10-3	0.145
Ag-90-8-3	0.072

**Table 2:** Content of gold in different ramie fibers treated with gold nanoparticles

Content of Au (wt%)		Content of Au (wt%)	
Au-90-5-2	0.0285	Au-90-4-6	0.0945
Au-90-5-4	0.0536	Au-90-3-6	0.0945
Au-90-5-6	0.0832	Au-90-2-6	0.0945
Au-90-5-8	0.117	Au-80-5-6	0.0857
Au-90-7-2	0.0254	Au-70-5-6	0.0866
Au-90-7-4	0.0492	Au-60-5-6	0.0902
Au-90-7-6	0.0700	Au-50-5-6	0.0887
Au-90-7-8	0.0906	Au-40-5-6	0.0572
Au-90-6-6	0.0764		

Color fastness of dyed fabrics is important in practical applications. The same as ramie fibers, the ramie fabrics were colored by in situ synthesized silver and gold nanoparticles. The color fastness to washing of the ramie fabrics treated with noble metal nanoparticles was evaluated. The ramie fabrics were washed for 45 min in the presence of ECE reference detergent at 50 °C under each washing cycle. The color differences (ΔE) of the ramie fabrics before and after washing are shown in Additional file 1: Figure S5. The visible color difference of silver nanoparticle-treated ramie fabrics after the first washing cycle was found with ΔE of 5.2 (Additional file 1: Figure S5). However, the average ΔE value of fabrics treated with silver nanoparticles did not increased notably with further increase in number of washing cycles. Compared with silver nanoparticle-treated ramie fabrics, the gold nanoparticle-treated ramie fabrics showed very good washing color fastness. The average ΔE value of fabrics treated with gold nanoparticles was 2.0 after first washing cycle (Additional file 1: Figure S5), which corresponds to the gray scale rating of 4. The color difference of gold nanoparticle-treated fabrics changed slightly and remained stable as the number of washing cycles increased, which indicates that the ramie fabrics colored by in situ synthesized gold nanoparticles exhibited good washing color fastness.

**Fig. 6:**
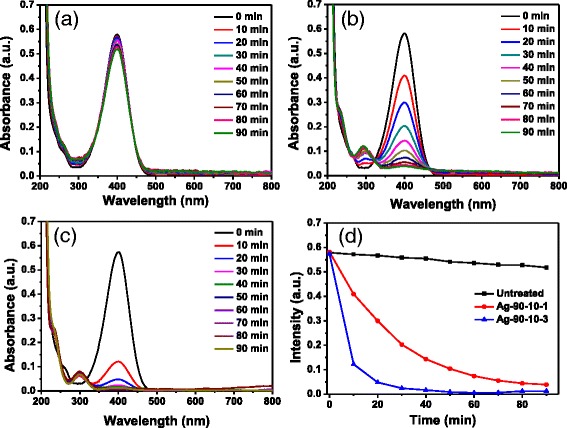
Evolution of UV-vis absorption spectra of 4-nitrophenol solution with **a** untreated ramie fibers, **b** Ag-90-10-1, and **c** Ag-90-10-3 after NaBH_4_ solution was added. **d** Plots of corresponding band intensity at 400 nm as a function of reaction time

Silver and gold nanoparticles have been used as catalysts for the reduction reactions of nitrophenols, nitroanilines, and dyes [28–31]. In this study, silver and gold nanoparticles were combined with ramie fiber through in situ synthesis reaction. Ramie fiber as a supporter of nanoparticles facilitates the separation of silver and gold nanoparticles from catalyzed reaction system after the reaction is finished, allowing the catalysts to be reused. The catalytic activity of ramie fibers treated with noble metal nanoparticles was evaluated using the reduction of 4-nitrophenol (4-NP) by sodium borohydride (NaBH_4_) as a model reaction. Nitro compounds are inert to NaBH_4_ without a catalyst [32, 33]. However, metal nanoparticles can catalyze the reaction by acting as an electronic relay agent to transfer electron from NaBH_4_ to the nitro compounds [32, 34]. The color of 4-nitrophenol changed to green yellow from light yellow after NaBH_4_ was added. Meantime, the absorption peak of 4-nitrophenol solution shifted to 400 nm after addition of NaBH_4_ due to the formation of 4-nitrophenolate ions [34–36]. Figure 6a–c shows the time-resolved UV-vis absorption spectra of 4-nitrophenol solution mixing with NaBH_4_ in the presence of untreated ramie fibers and silver nanoparticle-treated ramie fibers (Ag-90-10-1 and Ag-90-10-3), respectively. The absorption peak at 400 nm of 4-nitrophenol decreased slightly in intensity corresponding to untreated ramie (Fig. 6a). Nevertheless, the intensity of absorption peak at 400 nm of 4-nitrophenol solution with silver nanoparticle-treated ramie fibers decreased distinctly as reaction time prolonged after NaBH_4_ was added (Fig. 6b, c). Meantime, a new absorption peak at 300 nm appeared in the reduction process of 4-nitrophenol, which indicates the formation of 4-aminophenol (4-AP) [33, 34]. The change of the intensity of characteristic peak (400 nm) can indicate the reaction rate of reduction of 4-nitrophenol. Figure 6d depicts the plots of the corresponding absorption peak intensity at 400 nm as a function of time. As can be seen, the absorption intensity at 400 nm of 4-nitrophenol solution with untreated ramie fibers did not change obviously, revealing the untreated ramie fibers do not have catalytic activity for reduction of 4-nitrophenol. However, the absorption intensity at 400 nm corresponding to silver nanoparticle-treated ramie fibers (Ag-90-10-1 and Ag-90-10-3) changed dramatically, which suggests that the ramie fibers with silver nanoparticles show significant catalytic activity for reduction of 4-nitrophenol by NaBH_4_. It was noted that change rate of peak intensity corresponding to Ag-90-10-3 is higher than that corresponding to Ag-90-10-1 (Fig. 6d), which may be due to higher silver content of Ag-90-10-3 than Ag-90-10-1. These results demonstrate that ramie fibers treated with in situ silver nanoparticles have remarkable catalytic activity for reduction of 4-nitrophenol.

**Fig. 7:**
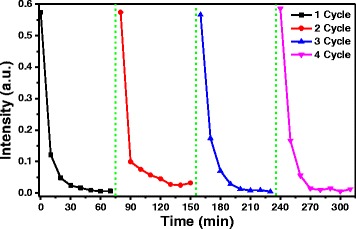
Recycling and reuse of silver nanoparticle treated ramie fibers (Ag-90-5-3) for the reduction of 4-nitrophenol to 4-aminophenol (4-AP)

Silver nanoparticle-treated ramie fibers were separated readily from reaction system. The treated fibers were applied for catalysis reaction again to examine the reusability of the treated fibers. Figure 7 displays four cycles of use of silver nanoparticle treated ramie fibers (Ag-90-10-3) for reduction of 4-nitrophenol. The catalytic activity of treated fibers did not decrease visibly even after the fourth cycle, which testifies that the treated ramie fibers as a catalysis could keep durable catalysis property.

In addition to silver nanoparticle-treated ramie, catalysis ability of the fibers treated with gold nanoparticles was investigated as well. The absorption peak at 400 nm of 4-nitrophenol decreased gradually in the presence of gold nanoparticle-treated ramie fibers (Au-90-5-6) with reaction time (Additional file 1: Figure S6a). The plot of peak intensity at 400 nm versus time proves that the ramie fibers with gold nanoparticles exhibit evident catalytic activity (Additional file 1: Figure S6b). The results indicate that the ramie fibers with noble metal (silver and gold) nanoparticles show effective catalysis cavity.

Silver nanoparticles as antibacterial agents have been used widely for antibacterial finishing of textile products [12, 37]. In the present study, the antibacterial properties of silver nanoparticle-treated ramie fibers were evaluated against the Gram-negative bacteria, *E. coli*. Additional file 1: Figure S7 shows the photographs of the bacterial colonies on blank (control) sample, untreated ramie fibers, silver nanoparticle-treated fibers (Ag-90-10-3). Plenty of colonies of viable bacteria were seen on agar plates corresponding to the control sample and untreated fibers (Additional file 1: Figure S7a, b). Whereas, no bacteria colonies were found on agar plates of ramie fibers treated with silver nanoparticles (Additional file 1: Figure S7c), revealing that the silver nanoparticles on fibers inhibited the growth of bacteria. Therefore, it is suggested that the in situ synthesized silver nanoparticles endow ramie fiber with strong antibacterial property, which enhances the function of ramie fibers.

Additional file 1: Figure S8 displays the ^13^C solid-state NMR spectra of ramie fibers before and after treatment with noble metal nanoparticles. It is well-known that the NMR technique probes the signal from the overall sample volume and the NMR intensity is proportional to the number of spins. Therefore, for the present ramie samples, the NMR spectra provide us the structure and dynamics information of the bulk molecules rather than the surface because of its small surface-to-volume ratio. The NMR spectra corresponding to untreated and treated ramie fibers were nearly identical, which indicates that the in situ synthesis of noble metal nanoparticles did not change visibly the chemical structures of the bulk ramie. As an indispensable compensation to NMR, FTIR detects the structure and dynamics of the surface molecules. To understand the surface structure change of the treated ramie fibers, FTIR measurements were performed for all the three samples. Comparing with the FTIR spectrum of untreated ramie (Curve a in Additional file 1: Figure S9), no obvious differences were found in the FTIR spectra of the fabrics after treatment with silver and gold nanoparticles (Curves b and c in Additional file 1: Figure S9). These results suggest that there is no evidence of degradation on the surface of ramie fibers. The main chemical structures of the surface of ramie fibers kept essentially unchanged during the in situ synthesis of silver and gold nanoparticles, which is consistent with the cases of cotton and bamboo treated with noble metal nanoparticles [16, 38]. The ramie is composed mainly of cellulose [39–41]. Cellulose as a reducing agent has been used for synthesis of silver and gold particles because of the presence of hydroxyl groups [42]. Additionally, other components of ramie including lignin, gum, and pectin may reduce silver or gold ions to form nanoparticles, due to their reducibility [43–45]. Further investigation on the mechanism for the reduction of silver and gold ions by ramie is in progress.

## Conclusions

Silver and gold nanoparticles were in situ synthesized on ramie fibers at different pH values through heating. Alkaline condition is required to synthesize silver nanoparticle in the presence of ramie. Whereas, acidic condition facilitated the synthesis of gold nanoparticles on ramie fibers. The formation of silver and gold nanoparticle imparted bright colors to ramie fibers. The color strength (K/S) of ramie fibers increased with concentration of precursor ions in solution. UV-vis reflectance absorption spectroscopy and SEM demonstrated that silver and gold nanoparticles were produced on ramie fibers. The contents of silver or gold were analyzed under different conditions. Significantly, the ramie fibers treated with in situ synthesized silver and gold nanoparticles showed remarkable catalytic activity for reduction of 4-nitrophenol. The treated ramie fibers could be separated readily and reused in catalyzed reaction. The ramie fabrics treated with gold nanoparticle had good color fastness to washing. Additionally, the silver nanoparticle-treated fibers showed significant antibacterial property. The finishing of ramie fibers based on the in situ synthesis of noble metal nanoparticles would pave the way for the development of functional ramie fibers.

## Additional File

Additional file 1: **Electronic supplementary information (ESI).** The file contains supplementary Tables S1–S6 and Figures S1–S9, and NMR testing procedure. (DOC 2501 kb)
