# Supplementary material for: Functional Application of Noble Metal Nanoparticles In Situ Synthesized on Ramie Fibers
Source: Nanoscale Res Lett. 2015 Sep 17;10:366. doi: 10.1186/s11671-015-1074-1 (PMC4574039; doi:10.1186/s11671-015-1074-1)
Supplement: Supplementary file 1 — Electronic supplementary information (ESI). The file contains supplementary Tables S1–S6 and Figures S1–S9, and NMR testing procedure. (DOC 2501 kb) [file 11671_2015_1074_MOESM1_ESM.doc]

Supplementary Information

Functional Application of Noble Metal Nanoparticles In-Situ Synthesized on Ramie Fibers

Bin Tang^1,2*^, Ya Yao^1^, Jingliang Li^2^, Si Qin^2^, Haijin Zhu^2^, Jasjeet Kaur^2^, Wu Chen^1^, Lu Sun^1,2^, Xungai Wang ^1,2*^

^1^School of Textile Science and Engineering, Wuhan Textile University, Wuhan 430073, China.

^2^Institute for Frontier Materials, Deakin University, Geelong, Victoria 3216, Australia.

^*^ Correspondence: bin.tang@deakin.edu.au; xungai.wang@deakin.edu.au.

**Table S1** Details of the experimental condition for in-situ synthesis of silver nanoparticles.

| pH value | Concentration of silver ions | | | | Temperature |
| --- | --- | --- | --- | --- | --- |
|  | 0.1 mM | 0.2 mM | 0.3 mM | 0.4 mM |  |
| pH=10 | Ag-90-10-1 | Ag-90-10-2 | Ag-90-10-3 | Ag-90-10-4 | 90 ^o^C |
| pH=7 | Ag-90-7-1 | Ag-90-7-2 | Ag-90-7-3 | Ag-90-7-4 |  |

**Table S2** Details of the experimental condition for in-situ synthesis of gold nanoparticles.

| pH value | Concentration of gold ions | | | | Temperature |
| --- | --- | --- | --- | --- | --- |
|  | 0.02 mM | 0.04 mM | 0.06 mM | 0.08 mM |  |
| pH=5 | Au-90-5-2 | Au-90-5-4 | Au-90-5-6 | Au-90-5-8 | 90 ^o^C |
| pH=7 | Au-90-7-2 | Au-90-7-4 | Au-90-7-6 | Au-90-7-8 |  |

**Figure S1** Plots of maximum K/S value as a function of concentration of **a** AgNO_3_ and **b** HAuCl_4_ in solution corresponding to Fig. 4.

**Table S3** Details of the experimental condition for in-situ synthesis of silver nanoparticles at different temperatures.

| pH value | Temperature | | | | | | Concentration  of  silver ions |
| --- | --- | --- | --- | --- | --- | --- | --- |
|  | 40 ^o^C | 50 ^o^C | 60 ^o^C | 70 ^o^C | 80 ^o^C | 90 ^o^C |  |
| pH=10 | Ag-40-10-3 | Ag-50-10-3 | Ag-60-10-3 | Ag-70-10-3 | Ag-80-10-3 | Ag-90-10-3 | 0.3 mM |

**Table S4** Details of the experimental condition for in-situ synthesis of gold nanoparticles at different temperatures.

| pH value | Temperature | | | | | | Concentration  of  gold ions |
| --- | --- | --- | --- | --- | --- | --- | --- |
|  | 40 ^o^C | 50 ^o^C | 60 ^o^C | 70 ^o^C | 80 ^o^C | 90 ^o^C |  |
| pH=5 | Au-40-5-6 | Au-50-5-6 | Au-60-5-6 | Au-70-5-6 | Au-80-5-6 | Au-90-5-6 | 0.06 mM |

**Figure S2** **a** K/S curves of ramie fibers with silver nanoparticles obtained with 0.3 mM of AgNO_3_ and pH = 10 at different temperatures. **b** Plot of maximum K/S value of silver nanoparticle treated ramie fibers as a function of temperature.

**Figure S3 a** K/S curves of ramie fibers with gold nanoparticles obtained with 0.06 mM of HAuCl_4_ and pH = 5 at different temperatures. **b** Plot of maximum K/S value of gold nanoparticle treated ramie fibers as a function of temperature.


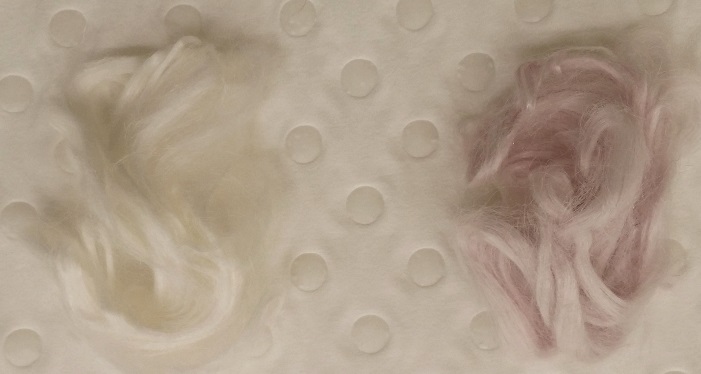


**Figure S4** Photograph of ramie fibers treated in (Left) AgNO_3_ (0.3 mM) and (Right) HAuCl_4_ (0.06 mM) solutions at pH = 7 and 90 ^o^C.

**Table S5** Details of the experimental condition for in-situ synthesis of silver nanoparticles at different pH values.

| Temperature | pH value | | | | | | Concentration of silver ions |
| --- | --- | --- | --- | --- | --- | --- | --- |
|  | pH=7 | pH=8 | pH=9 | pH=10 | pH=11 | pH=12 |  |
| 90 ^o^C | Ag-90-7-3 | Ag-90-8-3 | Ag-90-9-3 | Ag-90-10-3 | Ag-90-11-3 | Ag-90-12-3 | 0.3 mM |

**Table S6** Details of the experimental condition for in-situ synthesis of gold nanoparticles at different pH values.

| Temperature | pH value | | | | | | Concentration of gold ions |
| --- | --- | --- | --- | --- | --- | --- | --- |
|  | pH=2 | pH=3 | pH=4 | pH=5 | pH=6 | pH=7 |  |
| 90 ^o^C | Au-90-2-6 | Au-90-3-6 | Au-90-4-6 | Au-90-5-6 | Au-90-6-6 | Au-90-7-6 | 0.06 mM |


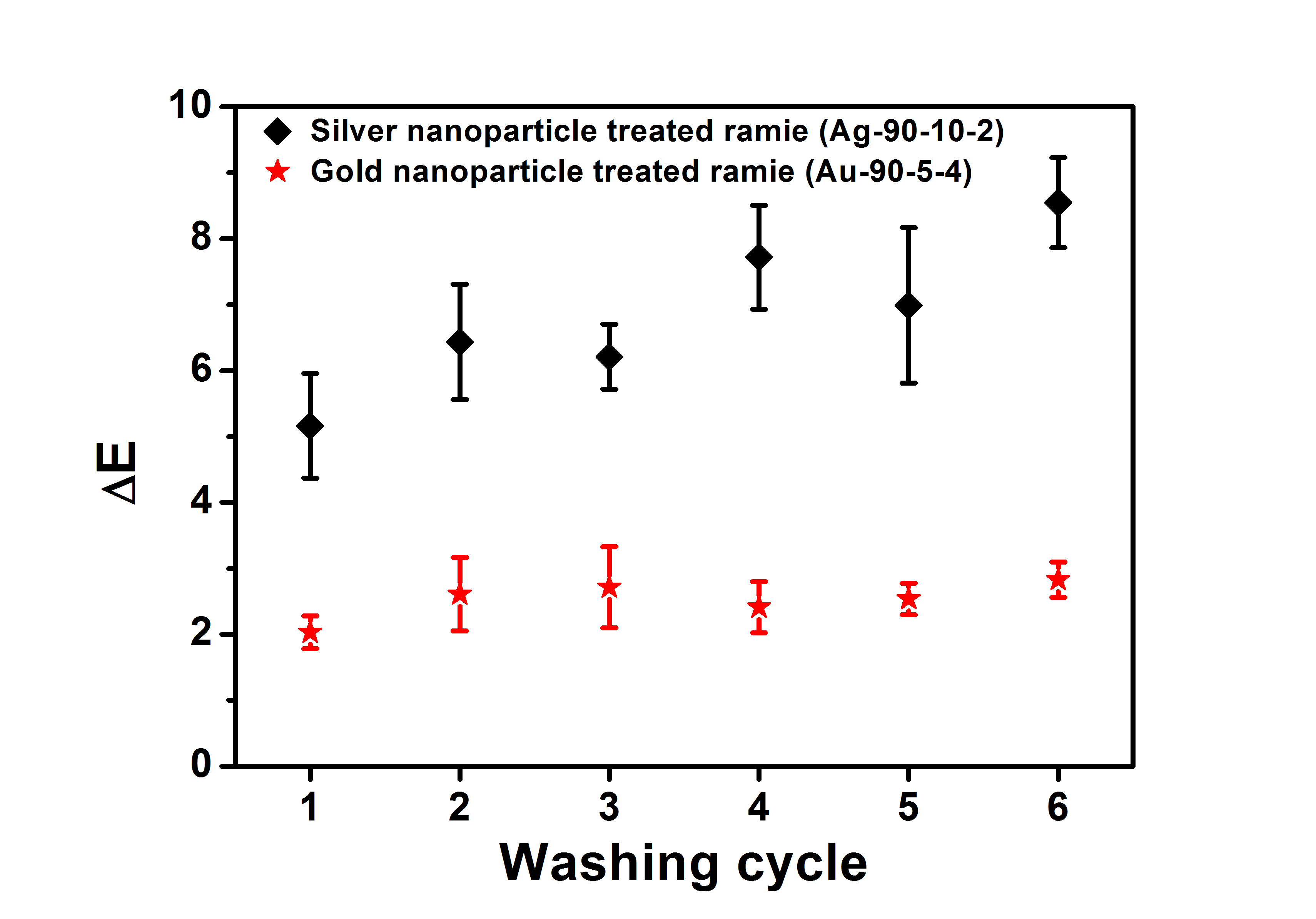


**Figure S5** Evolution of the color difference (ΔE) of ramie fabrics with in-situ synthesized silver and gold nanoparticles as the number of washing cycles increased.

**Figure S6** **a** Evolution of UV-vis absorbance spectra of 4-NP solution with Au-90-5-6 after addition of NaBH_4_ solution. **b** Plots of band intensity at 400 nm as a function of reaction time corresponding to untreated fiber and Au-90-5-6.

**Figure S7** Antibacterial activity measurement of **a** blank sample (control), **b** untreated bamboo pulp fabric, **c** silver nanoparticle treated ramie fibers.

**Figure S8** ^13^C solid-state CP-MAS spectra of the untreated, silver nanoparticle treated and gold nanoparticle treated ramie fibers.

**Figure S9** FTIR spectra of untreated, silver nanoparticle treated and gold nanoparticle treated ramie fibers.

**Nuclear magnetic resonance (NMR) testing**

^13^C solid-state NMR experiments were performed on a Bruker Avance III 500 MHz wide bore NMR spectrometer (^1^H Lamor frequency of 500.07 MHz) equipped with a 2.5 mm double resonance Magic Angle Spinning (MAS) probe head. ^13^C cross polarization (CP)-MAS NMR spectra were recorded with a MAS rate of 25 kHz. A 75 kHz ^1^H continuous wave (CW) high power decoupling was applied during acquisition to eliminate the ^1^H-^13^C dipole-dipole broadening and to enhance the ^13^C signal. The use of 10240 scans was applied for each sample, with a recycle delay of 2s.
